# Identification and Determination of Synthetic Pharmaceuticals as Adulterants in Eight Common Herbal Weight Loss Supplements

**DOI:** 10.5812/ircmj.15344

**Published:** 2014-03-05

**Authors:** Marjan Khazan, Mehdi Hedayati, Farzad Kobarfard, Sahar Askari, Fereidoun Azizi

**Affiliations:** 1 Endocrine Research Center, Research Institute for Endocrine Sciences, Shahid Beheshti University of Medical Sciences, Tehran, IR Iran; 2 Cellular and Molecular Endocrine Research Center, Research Institute for Endocrine Sciences, Shahid Beheshti University of Medical Sciences, Tehran, IR Iran; 3 Department of Medical Chemistry, School of Pharmacy, Shahid Beheshti University, Tehran, IR Iran

**Keywords:** United States Food and Drug Administration, Herbal Medicine, Weight Loss, Gas Chromatography-Mass Spectrometry

## Abstract

**Background:**

Adulterated herbal weight loss products with containing undeclared synthetic drugs are common and responsible for many serious health damages.

**Objectives:**

The purpose of the study was to determine five synthetic adulterants in eight common herbal weight loss supplements, which are currently sold in Iran markets, to verify their presence in supplements, without mentioning on the labels.

**Materials and Methods:**

Eight common herbal weight loss samples were obtained from the Iran pharmaceutical market after advertising in the Persian language on satellite channels and internet. Five pharmacological classes of drugs used for weight loss, namely sibutramine, phenolphthalein, phenytoin, bumetanide and rimonabant, were investigated and quantified by GC-MS for the first three and LC-MS for the last two medications.

**Results:**

The most undeclared ingredients, which were illegally added include sibutramine, phenolphthalein, bumetanide, and phenytoin in the original super slim, herbaceous essence, super slim green lean, and fat loss, supplements, respectively. Rimonabant was not found. Caffeine, pseudoephedrine, theobromine and amfepramone were also found in the supplements using GC-MS assay.

**Conclusions:**

Adulterated synthetic substances were detected in the herbal weight loss products. Health care professionals should make people aware of the risks of taking herbal weight-loss supplements.

## 1. Background

Nowadays the use of herbal weight loss products as alternative obesity treatment has increased, which leads to the growing market of herbal remedies worldwide (1, 2). According to the consumer’s belief, these products are “natural herbal components” and are regarded as harmless and effective methods of obesity treatment (3, 4). Since these adulterated herbal products are manufactured illegally, their safety, efficacy, and quality control are not verified (5, 6). The presence of synthetic substances and the analogues of prescription drugs in adulterated herbal weight loss formulations, can cause multiple health risks, that pose major concerns for all health agencies around the world (3, 7, 8). Thus, the World Health Organization (9), Food and Drug Administration (FDA), European Medicines Agency (10) have issued several guidelines for safe and appropriate use of herbal medicines (11, 12). De Carvalho et al. (5) showed that the most probable adulterant classes of pharmaceuticals used in weight loss formulations are anorexic (sibutramine, rimonabant), anxiolytic (benzodiazepines), antidepressant (fluoxetine.), diuretic (furosemide) and laxative (phenolphthalein). In addition the presence of other pharmaceutical classes, such as ephedrine, bumetanide (13), phenytoin, caffeine and thyroid hormones in weight loss formulations has been recently reported in the Netherlands (14), UK (Royal College of Physicians 1998), USA (15), and Iran (16). Unfortunately, consumers who took adulterated herbal weight loss supplements complained of many side effects and clinical problems such as, cardiovascular disease (15), liver (17, 18) and renal failure (5), mental/mood changes e.g. excitement, restlessness, confusion, depression and occasionally even thoughts of suicide (16, 19, 20). Herbal medicines are sold at herbal shops and also advertised by satellite channels in the Persian language or on internet with direct delivery (2). One problem is that, a herbalist (a person who involves in production, distribution and application of herbal remedies) usually does not have the appropriate knowledge or the required ethics (3, 5). Marketing and sale of these supplements on the internet and satellite channels are not strictly controlled and are not always required to pass safety and control tests before advertisement and sell in the market.

## 2. Objectives

Considering the above mentioned, we investigated and quantified five synthetic adulterants, namely sibutramine, phenolphthalein, phenytoin, bumetanide and rimonabant in eight common adulterated herbal weight loss products which are currently sold in Iran market.

## 3. Materials and Methods

### 3.1. Samples

Eight samples suspected for adulteration with undeclared synthetic drugs were obtained from herbalists and markets. These products were mainly from China and some Southeast Asia countries, and had been declared as natural herbal mixtures, which were in the form of pills (Mobic, Shangaya HG) or capsules (Magic Slim, Green lean Super Slim, Original Super Slim, Fast Slim, Herbaceous essence, Fat loss). The recommended dosage by the manufacturer was 2 - 3 capsules or pills per day. These common herbal weight loss supplements were analyzed using GC-MS and LC-MS methods; the GC-MS method was used for the screening of sibutramine, phenolphthalein, and phenytoin while the LC-MS method was used for the analysis of rimonabant and bumetanide.

### 3.2. Chemicals

Authenticated standards of sibutramine, phenolphthalein, bumetanide, and phenytoin were obtained by Sigma-Aldridch and rimonabant was also provided by MolPort-003-850-185. Methanol used in this investigation had the analytical grade purity and was purchased from Merck.

### 3.3. Sample Preparation

One capsule or pill was randomly selected from each product. Capsules were directly emptied for weighing, whereas pills were ground before weighing. Of each homogenous powder, 5 mg was extracted with 10 mL methanol for 30 minutes in round bottom test tube using laboratory rotator. The extract was centrifuged for 10 minutes at 8000 rpm; then filtered. The supernatant was collected for examination. The linear calibration range for all the compounds was between 1 - 500 µg/mL. Lower limit of quantifications (LLOQ) for all the compounds were obtained as 5 ng/mL and no further attempts were made to improve. All of the compounds except bumetanide had excellent accuracy (average of 95.6 %) and precision (average of 6.7 % RSD). Bumetanide had lower accuracy (average of 87.2 %) and precision (11 % RSD).

### 3.4. Measurements

The samples were analysed for detection of rimonabant and bumetanide using the liquid chromatography/mass spectrometry analysis (LC–Mass) method; the LC–Mass system consisted of an Agilent 1200 series HPLC coupled with an Agilent 6410 series triple quadrupole mass spectrometer with an electrospray ionization interface (Agilent Technologies Inc., CA, USA). In the LC assay, the Mobile phase was methanol/water solutaion (90:10) containing ammonium formate buffer (2mM, pH = 2.4), flow rate 0.35 mL/min. The column was a C-18 Zorbax column and the column oven temperature adjusted at 55 °C; mass spectrometer was operating at positive mode. Drying gas temperature was 350 °C, Nebulizer 30 psi, drying gas flow rate was 12 L/min. Data collection was performed over the mass range of 100 to 1000; capillary voltage was 30000 V and fragmentation voltage was adjusted at 35V.

In order to determine the adulterants, i.e. sibutramine, phenolphthalein, bumetanide, and phenytoin in the tablets or capsules, the GC/MS (Agilent 7000, Triple Quaed, GC7890A) was used. A capillary column [HP-5ms, 30 m (length), 0.25 mm (diameter), 0.25 μm (film)] was used as the stationary phase. The column oven temperature was initially held at 50°C for two minutes, and then increased to 290°C at the ramp rate of 5 °C/min. and held for 10 minutes at the same temperature. The total run time was 36 minutes. The temperatures of the injector and detector were set at 250 and 300°C, respectively. The carrier gas was helium, at a working flow rate of 1 mL/min and the injection volume was 1 μL. The MS conditions were: ionization energy 70 eV, mass range 25 - 1000 amu and ionization technique was electron impact. The identification of constituents in our samples was carried out by comparing their retention time and their mass spectra with those of pure reference standards and correspondent drugs found on the market. Mass spectra were also compared with those in the Wiley 275 and NIST libraries installed on the GC-MS linked computer.

## 4. Results

**Figure 1.**
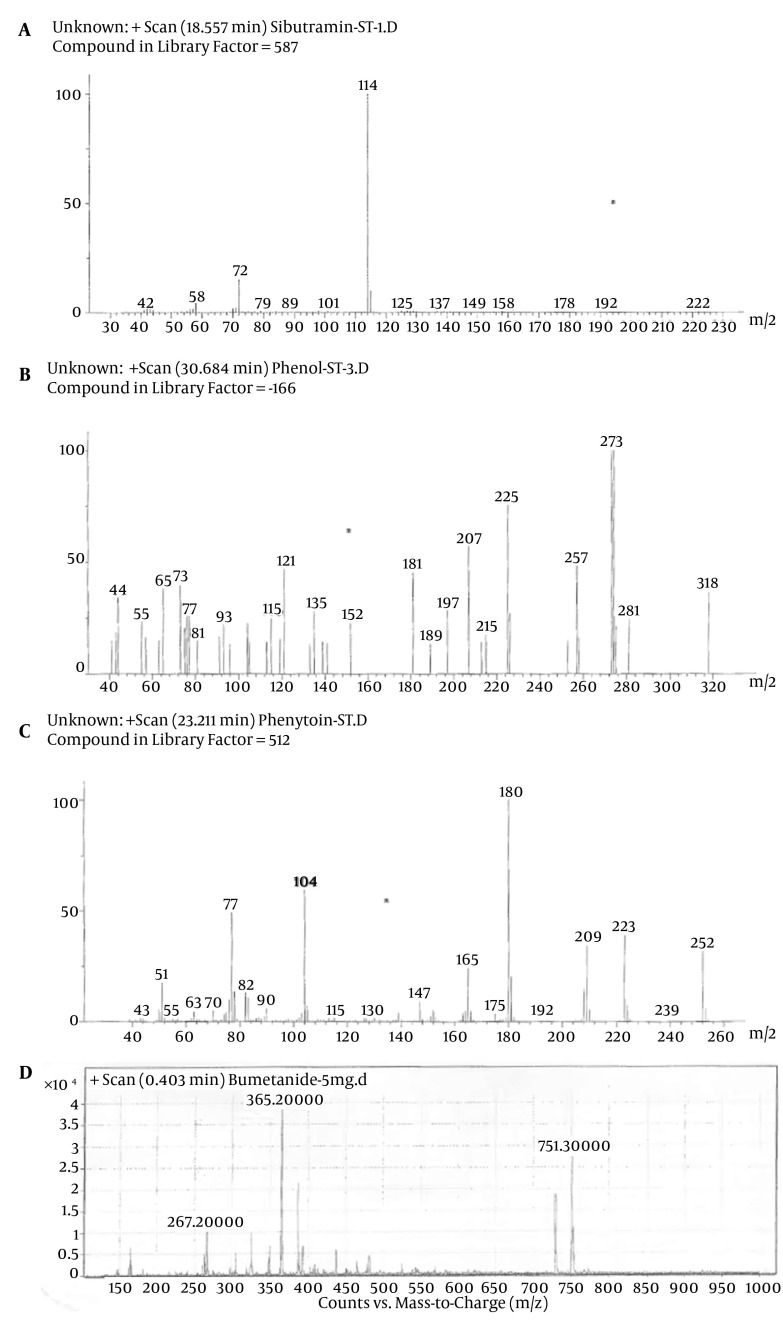
The Mass Spectra of Standard Solutions of Sibutramine (a), Phenolphthalein (b), and Phenytoin (c) Obtained by GC-MS and Bumetanide (d) Obtained by LC-Ms

**Figure 2.**
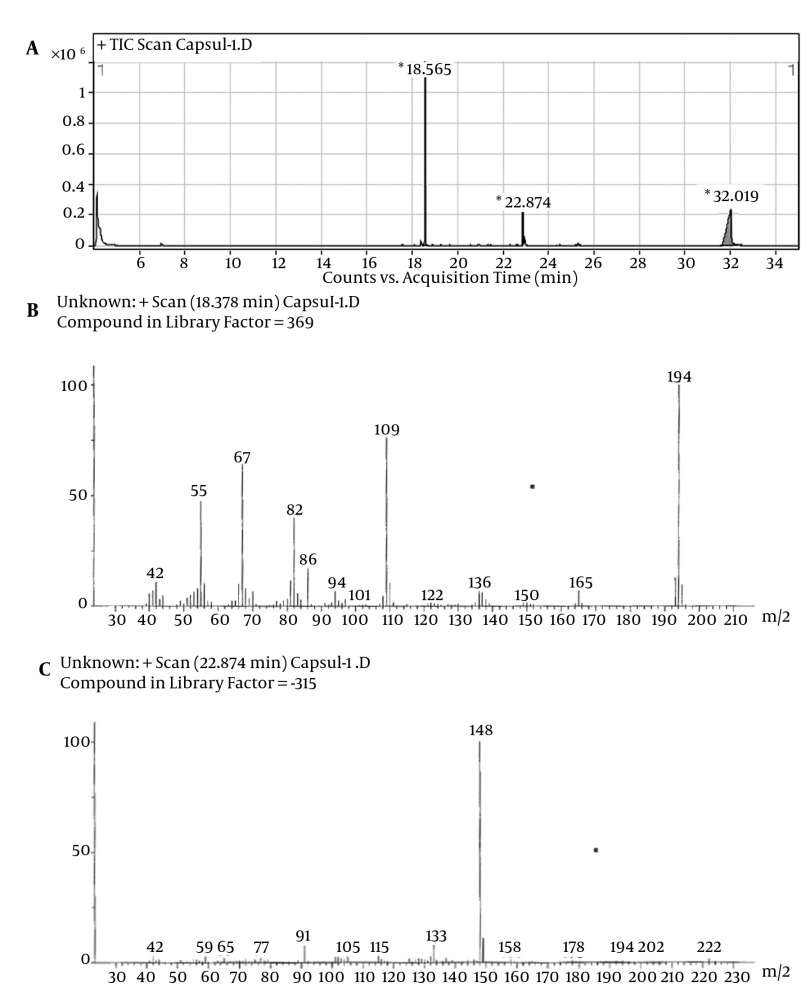
The Chromatogram of Herbaceous Essence (a) by GC-MS and the Mass Spectra Obtained for Caffeine (b), Pseudoephedrine (c) and Theobromine (d)

**Figure 3.**
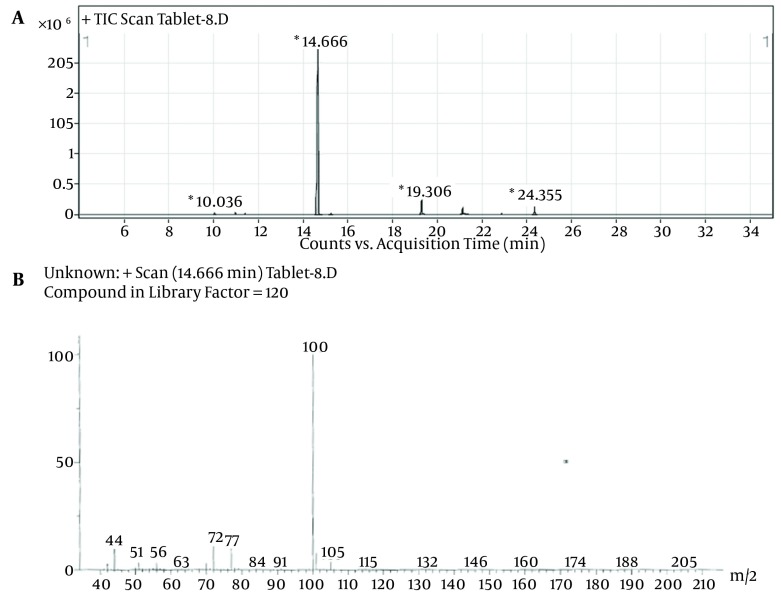
The Chromatogram of Fat Loss (a) By GC-MS and the Mass Spectra Obtained for Amfepramone (b)

Despite the manufacturer’s claim that their products contained only the extracts of the plants mentioned on the label, but those are comprised of other synthetic substances (Table 1). Sibutramine, phenolphthalein, bumetanide and phenytoin were detected in six, three, five and two herbal products, respectively; caffeine, pseudoephedrine, theobromine and amfepramone were also found, based on a library search on Wiley 275 and NIST libraries, in four, one, three and one of the herbal weight loss remedies. Rimonabant was not found. Identification was performed based on retention time and molecular ions observed in EI-Mass spectra and pseudomolecular ions in ESI-Mass. The mass spectra of standard solutions of sibutramine (a), phenolphthalein (b), and phenytoin (c) were obtained by GC-MS and bumetanide (d) was obtained by LC-Ms (Figure 1). The chromatogram obtained for Herbaceous essence (a) by GC-MS and the mass spectra obtained for caffeine (b), pseudoephedrine (c) and theobromine (d) are shown in Figure 2 ; also in Figure 3,the chromatogram obtained for Fat loss (a) by GC-MS and the mass spectra for amfepramone (b) are presented.

**Table 1.** Adulterants Found in Eight Analyzed Herbal Weight Loss Products

Name of Products	Quantitative	Adulterants, Dose (mg/capsule or mg/pill)	Component
**Herbaceous Essence**	Sibutramine	30	Caffeine
	Phenolphthalein	1167	Pseudoephedrine
	Bumetanide	2.3	-
	phenytoin	0.1	-
**Magic Slim**	Sibutramine	6	Caffeine
	Phenolphthalein	233.8	-
	Bumetanide	2	-
**Green Lean Super Slim**	Sibutramine	15	-
	Bumetanide	3.8	-
**Original Super Slim**	Sibutramine	78	-
	Phenolphthalein	825	-
**Fast Slim**	Sibutramine	57	-
**Fat Loss**	Sibutramine	46	Amfepramone
	Bumetanide	0.82	-
	phenytoin	0.86	-
	-	-	-
**Mobic**	Bumetanide	1.6	Caffeine
	-	-	Theobromine
**Shangaya HG**	-	-	Caffeine
	-	-	Theobromine

## 5. Discussion

The results of the study showed the presence of illegal synthetic substances in common herbal weight loss supplements sold in Iran market. Adulterants identified and determined in the assessed herbal weight loss supplements were sibutramine, phenolphthalein, bumetanide and phenytoin; caffeine, pseudoephedrine, theobromine and amfepramone, which were also qualitatively found as miscellaneous compounds in the supplements. All synthetic adulterants used in these supplements are not mentioned on labels and therefore the consumers are kept unaware of their side effects and health problems; they assume they are taking a natural product but are unaware of the dangerous complications that they could be exposed to; these products have been illegally imported without any licensed label confirming their safety and quality from the related Ministry of Health. The manufacturers of such illegal products add some synthetic pharmaceuticals as adulterants to their products to make them more efficacious for the consumers (10, 11, 14).

One adulterant is sibutramine often reported as the main undeclared adulterant in a herbal weight loss formula (5, 14, 21-23); its most common side-effects are psychotic and mood changes(16, 19, 24). Also, some studies showed sibutramine has been associated with increased cardiovascular outcomes, heart failure and sudden death, causing it to be withdrawn from the market in the United States, United Kingdom, European Union, Australia, Canada and some Asian countries (11, 25). However, In Iran, it is still prescribed as a medicine and the dose range of sibutramine is 5 -15 mg daily (26) which requires regular monitoring. On the other hand, the recommended dose for herbal weight loss pills are 2-3 tablets per day, which is higher than the therapeutic dose of sibutramine (Table 1). 

The next adulterant is the laxative phenolphthalein that was withdrawn as a medicine several years ago because of its carcinogenic effects (14, 27). Hence, high phenolphthalein intake increases the risk of cancer (Table 1); that is also associated with sibutramine in several herbal weight loss formulations (5, 14, 27). Bumetanide as a diuretic, like laxatives, is not very effective as a weight-loss drug but has similar health risks (4, 28). Although not prescribed as a medicine in Iran, athletes in Arizona use diuretics to decrease weight and as masking agents of doping (13). Phenytoin is found only in trace amounts in some of these illegal supplements. In our study the amount of phenytoin was below the safety dose but its simultaneous consumption with diuretics and sibutramine usually increases the risk of their potential harmful effects (14).

Caffeine, pseudoephedrine and theobromine were reported as anorectic for weight loss purposes (18, 29-31). Using caffeine in combination with other stimulants or taken in massive doses increases the risk of serious life-threatening or debilitating adverse effects such as hypertension, myocardial infarction, stroke, seizure, and death (18, 32, 33). Thus, these classes of pharmaceuticals should also be considered as illegal substances in herbal weight-loss formulations. In recent years, derivatives of xanthines (caffeine and theobromine) have increasingly attracted the attention as adulterated natural products, that are used in weight loss drugs and energy drinks. Adulteration, lack of standardization, incorrect preparation and/or dosage and inappropriate labeling are the most common problems with such herbal products (34, 35).

Another stimulant drug is amfepramone, which is used as an appetite suppressant in adulterated herbal supplements, despite worries that these drugs might cause serious heart and lung problems in some users (36), but doctors also think there is a possible link between amfepramone and a severe mental illness called psychosis, which has been reported in a case who became psychotic after taking this drug (37). There has been much legal action against this drug, and it has been taken off the market several times but unfortunately is available again. In theory, although physicians in the UK can prescribe amfepramone, most are not sure that these drugs work well enough to lessen the risk of side effects. Aamfepramone is not used by the National Health Service (NHS) (38). Thus, although illegal herbal products might contain safety doses of some legal synthetic drugs such as phenytoin, caffeine, and theobromine, they are not officially prescribed for weight loss. Limitations: Since anti-obesity drug analogues are not limited in these products, we were not able to analyze and discuss all adulterated herbal weight loss supplements in Iran market. The most important limitation of this study was no accessibility to the serum samples of consumers for detecting adulterants and the data obtained is only limited to the herbal weight loss supplements. 

Nowadays, the consumption of herbal weight loss formulations has increased vastly, due to misleading advertisements for obesity treatment on internet and the media; adulterated weight loss products which cause health problems, and the ministry of health should warn people about these products. The efficacy and safety of herbal weight loss drugs should be tested and strict government control and regulation of their marketing and sales on Iran market are recommended.
